# Transcriptional Responses of Resistant and Susceptible Wheat Exposed to Wheat Curl Mite

**DOI:** 10.3390/ijms22052703

**Published:** 2021-03-08

**Authors:** Mahnaz Kiani, Becky Bryan, Charles Rush, Adrianna Szczepaniec

**Affiliations:** 1 Thegreencell, Inc., 15810 Gaither Drive, Gaithersburg, MD 20877, USA; 2 Department of Plant Pathology, Texas A&M AgriLife Research, Amarillo, TX 79106, USA; rebecca.bryan@ag.tamu.edu (B.B.); crush@ag.tamu.edu (C.R.); 3 Department of Entomology, Texas A&M AgriLife Research, Amarillo, TX 79106, USA; aszczepaniec@tamu.edu or

**Keywords:** *Aceria tosichella* Keifer, *Triticum aestivum* L., plant–insect interaction, RNA-seq, plant resistance

## Abstract

(1) Background: The wheat curl mite (*Aceria tosichella* Keifer) is a key pest of wheat (*Triticum aestivum* L.) worldwide. While a number of wheat cultivars resistant to the mites have been employed to minimize the impact on the yield and quality of grain, little is known regarding the mechanisms underlying host plant resistance. Therefore, the goal of this study was to explore changes in transcriptome of resistant and susceptible wheat in order to quantify the molecular changes that drive host plant resistance. (2) Methods: Two varieties, wheat curl mite-susceptible (Karl 92) and wheat curl mite-resistant (TAM112) wheat, both at 2-week postemergence, were used in this study. Half of the plants were exposed to wheat curl mite herbivory and half remained mite-free and served as controls. Transcriptome changes were quantified using RNA-seq and compared among treatments to identify genes and pathways affected by herbivores. (3) Results: We identified a number of genes and pathways involved in plant defenses against pathogens, herbivores, and abiotic stress that were differentially expressed in the resistant wheat exposed to wheat curl mite herbivory but were unaffected in the susceptible wheat. (4) Conclusions: Our outcomes indicated that resistant wheat counteracts wheat curl mite exposure through effective induction of genes and pathways that enhance its defense responses.

## 1. Introduction

The wheat curl mite (WCM), *Aceria tosichella* Keifer (Acari: Eriophyidae), is one of the most significant pests of wheat (*Triticum aestivum* L.). WCMs are highly polyphagous, and their hosts include over 90 different species of grasses worldwide [1]. Their feeding reduces photosynthesis [2] and alters leaf morphology, resulting in the characteristic curling of leaves, particularly in seedling wheat [1]. The microscopic WCMs living within tightly curled leaves and whorls are effectively protected from pesticides, rendering already limited chemical control of them largely ineffective. Several biotypes of mites have been identified, and two of them (type 1 and type 2) are particularly widespread worldwide. Both biotypes can be found in mixed populations, even within a single wheat plant [3,4,5].

However, the main injury stemming from WCM is indirect, as viruses associated with this pest and transmitted to wheat account for millions of dollars in losses to wheat crop each year [6]. WCMs transmit Wheat streak mosaic virus (WSMV), High plains wheat mosaic virus (HPWMoV), Triticum mosaic virus (TriMV), and Brome streak mosaic virus (BrSMV) [7,8,9,10]. WSMV and TriMV are the most widespread among these viruses and have the greatest impact on wheat production, particularly in the Great Plains in the U.S. [1,11], while BrSMV appears to be limited to wheat grown in Europe and has little economic impact [8]. WCMs are the only known vectors of these viruses [12], and biotype 2 has been found to be a more efficient vector of WSMV than biotype 1 [13,14,15]. Notably, multiple viruses can be transmitted by mites to a single plant, and the coinfection of WSMV and TriMV can exacerbate disease symptoms and further reduce wheat yield [16,17,18]. Furthermore, WSMV appears to have a competitive advantage over TriMV owing to its positive effect on the survival and reproduction of WCMs [6]. In a recent study, Gupta et al. demonstrated that WSMV can manipulate WCM immune responses to its benefit. The authors reported that WSMV-infected mites develop faster than nonviruliferous WCMs largely due to the suppression of immune-related genes [19].

*Triticum* has low levels of innate resistance to WCMs or viruses they transmit [20], and genetic sources of high-level resistance have been introduced through introgression from other grasses such as *Aegilops tauschii* Coss. [21,22,23,24], tall wheatgrass, *Agropyron elongatum* Host [25], and rye *Secale cereal* L. [26]. WCMs frequently have lower reproduction when feeding upon resistant wheat, which contributes to a reduction in WSMV spread [27,28]. Wheat varieties resistant to WCMs and viruses they transmit have been employed successfully in crop production [29,30,31,32], but their resistance to vectors and associated viruses is often incomplete [11]. Further, their efficacy in the field has been noted to decrease in recent decades [11,20,33]. Plant resistance to WCMs has been generally more challenging to employ in breeding for resistant lines [26], and it has been less consistent than the resistance to WSMV or TriMV [20]. It is notable that the molecular mechanisms underlying WCM–wheat interactions in context of host plant resistance are not well known. Exploring these would enhance basic knowledge of plant responses to WCMs and contribute to breeding efforts.

Previous research has examined the mechanisms of plant resistance to other mites of high economic importance, such as two spotted spider mites, *Tetranychus urticae* Koch (Acari: Tetranychidae), but little is known regarding the molecular-level interactions between WCMs and their host plants [34]. For an example, only a few genes conferring resistance against WCMs through antibiosis from relatives of wheat within Poaceae have been identified [24,35]. Moreover, currently used resistant wheat lines such as TAM112 have only partial resistance to mites [36,37], and the molecular mechanisms underlying the resistance of host plants to WCM remain largely unknown. Thus, the goal of this work was to explore the consequences of WCM herbivory to wheat, resistant to the mites, using RNA-seq technology. We hypothesized that resistant wheat, fed upon by WCMs, would exhibit a suite of gene expression changes, particularly in its defense-related pathways that would be starkly different from transcriptome responses in wheat susceptible to the mites. This is the first study to quantify transcriptome-level responses to WCMs in wheat.

## 2. Results and Discussion

### 2.1. Mapping Results and Differentially Expressed Genes (DEGs) in the Resistant and Susceptible Wheat Exposed to WCM Herbivory

The sequencing of 16 libraries generated 67.6 to 98.24 million reads from the individual samples, and 54.3 to 75.3 million reads were uniquely mapped to wheat reference genome *Triticum aestivum* v2.2. On average, 88% of the reads were mapped to exonic regions (Supplementary Table S1). We also mapped the DEGs to the newer version of the wheat genome (IWGSC RefSeq v.1.0), but owing to few significant differences between samples we selected to explore the outcomes of the experiment using an older version of the genome, i.e., *Triticum aestivum* v2.2. It is not clear why the use of the newer version of the genome did not generate significant results or if lack thereof is biologically significant. It is noteworthy, however, that the older version of the genome that we used to compare wheat transcriptomes in our study has also been used in recently published research, such as Wang et al. [38]. The mapping results obtained using wheat genome version IWGSC RefSeq v.1.0. are presented in supplemental files (Supplementary Table S2).

**Figure 1:**
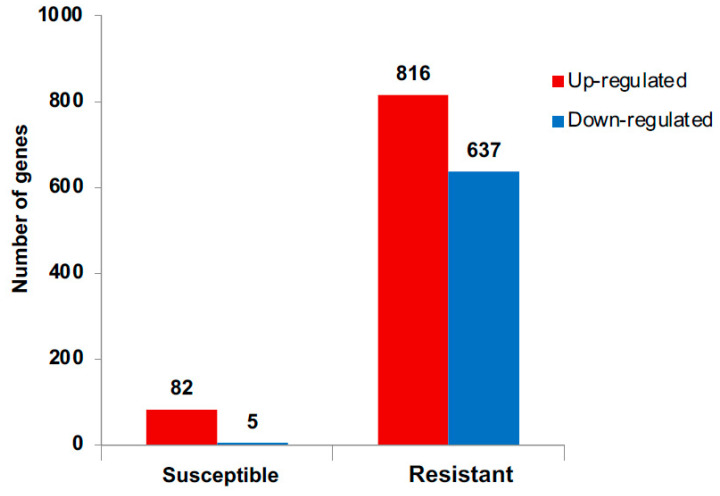
Numbers of differentially expressed genes (DEGs) in the susceptible and resistant wheat in response to wheat curl mite herbivory. DEGs were defined as having a fold change of ≥1.9 or ≤−1.9 with a false discovery rate (FDR)-adjusted *p*-value of <0.05.

**Figure 2:**
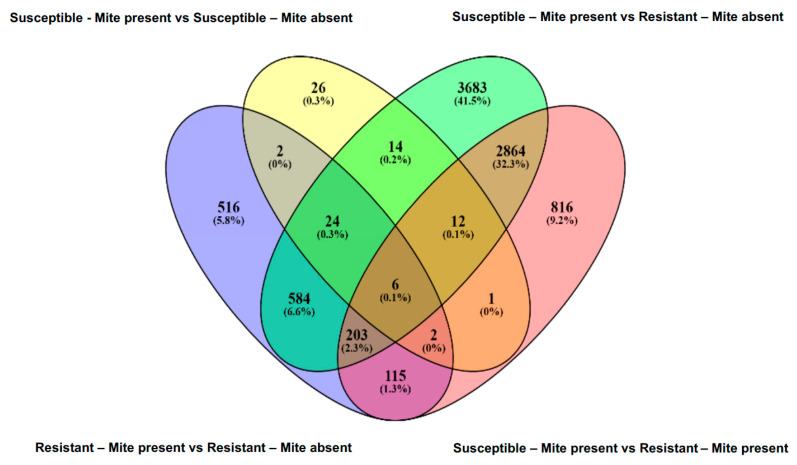
Venn diagram of DEGs in the susceptible and resistant wheat exposed to wheat curl mite herbivory.

The number of DEGs in response to WCM herbivory was much higher in the resistant genotype than in the susceptible wheat genotype (Figure 1). Of the 1453 DEGs in the resistant genotype, 56% were upregulated, and 44% were downregulated. On the other hand, only 87 DEGs were found in the susceptible genotype exposed to mite herbivory, of which the majority (94%) were upregulated and 6% were downregulated (Figure 1). The overlaps among sets of DEGs representing changes in the susceptible and resistant genotypes in response to WCM herbivory were further illustrated in Venn diagram (Figure 2). In total, WCM herbivory altered the expression of 34 genes that were shared between both genotypes, while 1418 and 53 DEGs were expressed uniquely in the resistant and susceptible genotypes, respectively. These results indicated that the resistant wheat responds to the mite feeding by eliciting a higher number of unique responses than the susceptible genotype. A higher number of DEGs in the resistant genotype in response to herbivory has been reported in several other species, such as in resistant sugarcane in response sugarcane borer, *Diatraea saccharalis* Fabricius (Lepidoptera: Pyralidae) infestation [39], and in sorghum in response to sugarcane aphid sugarcane aphid, *Melanaphis sacchari* Zehtner (Hemiptera: Aphididae) [40], suggesting that resistant plants undergo significantly greater metabolic changes, which may help them respond more effectively to herbivory by activating defense-related genes.

### 2.2. Metabolism Overview

**Figure 3:**
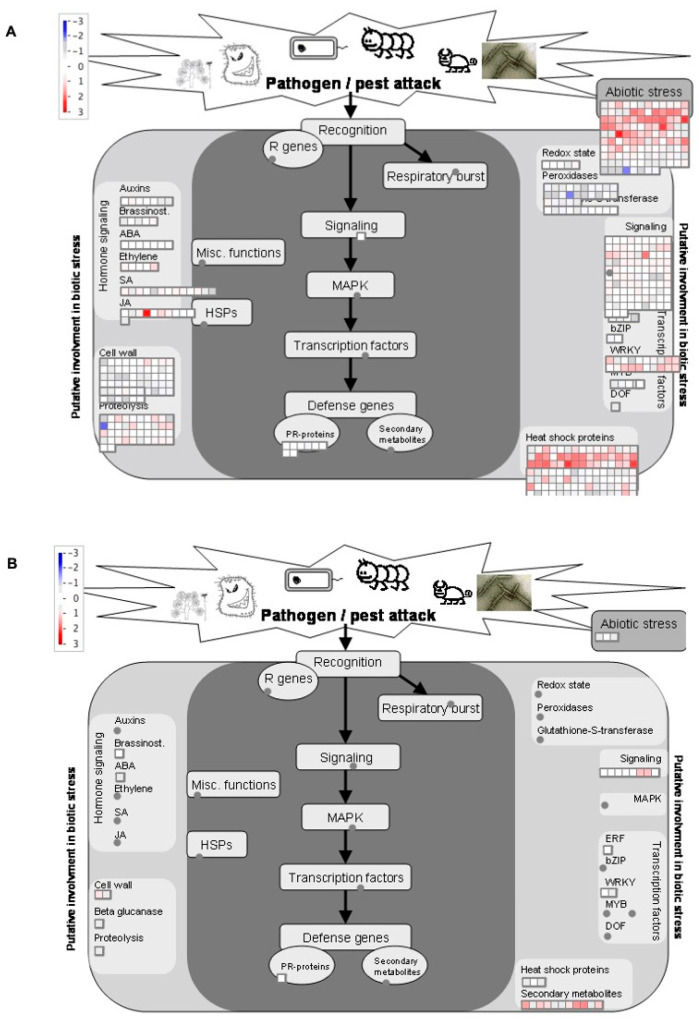
Overview of DEGs involved in response to wheat curl mite in the resistant (**A**) and susceptible (**B**) wheat varieties as visualized by MapMan. The colored boxes represent log10-transformed fold changes. The red color indicates increased expression in response to wheat curl mites, while the blue color indicates a decrease in expression.

In order to further characterize wheat responses to WCMs and visually represent the effects of wheat genotype on the induction of key pathways and processes, we used MapMan [41] (Figure 3). We detected a number of biological processes affected by mite herbivory in the resistant wheat that was not present in the susceptible genotype. We found that a high number of DEGs in the resistant wheat were involved in abiotic stress, hormone signaling, heat shock proteins, and gene transcription regulated by WKRY transcription factors. Further, the resistant wheat also showed gene expression changes related to secondary metabolism, biotic stress responses, cell wall composition, and abscisic acid (ABA) signaling. The numbers of these genes mapped to any of these categories were drastically lower or all together absent in the susceptible wheat. Such broad metabolic differences underlying host plant resistance have been noted previously in other systems. For example Reddy et al. reported profound transcriptomic changes in resistant, near isogenic, wheat lines compared to susceptible ones, at 24 and 48 h following infestations of plants with greenbug, *Schizaphis graminum* Rondani (Hemiptera: Aphididae) [42]. The authors reported the induction of transcripts belonging to several categories such as signaling, hormone metabolism, secondary metabolites, cell wall modification, peroxidases, and transcription factors. Niu et al. also reported an overall higher magnitude of metabolic changes in resistant peach lines compared to in susceptible line in response to green peach aphid, *Myzus persicae* Sülzer (Hemiptera: Aphididae) [43]. These examples along with the one presented in this study emphasize the scale of metabolic reshuffling that occurs in resistant plants and allows them to differentiate between the presence and absence of herbivores and respond accordingly.

### 2.3. Gene Ontology (GO) Analysis

**Figure 4:**
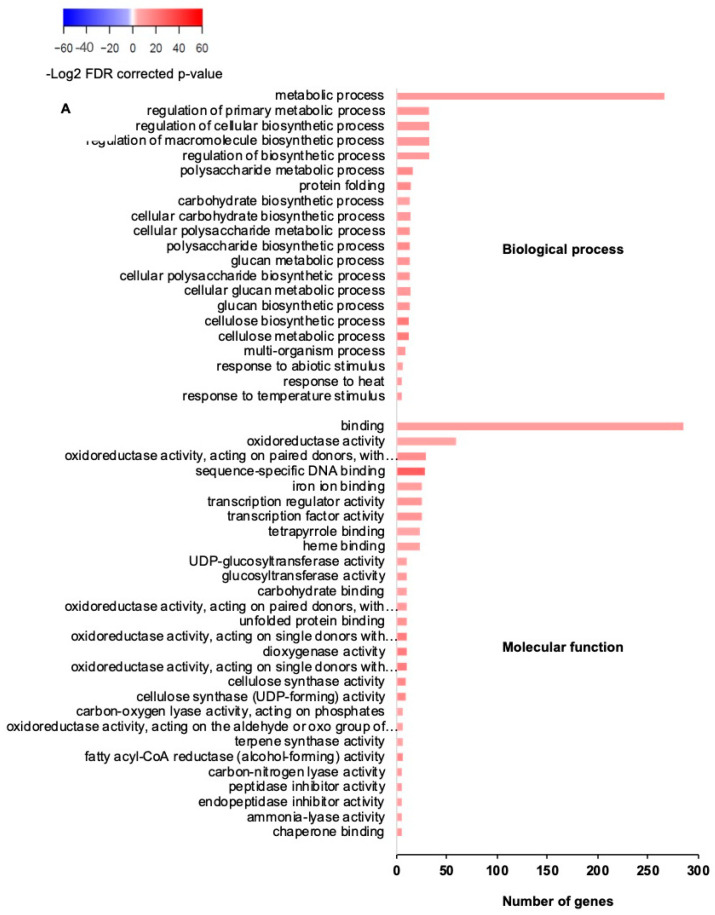

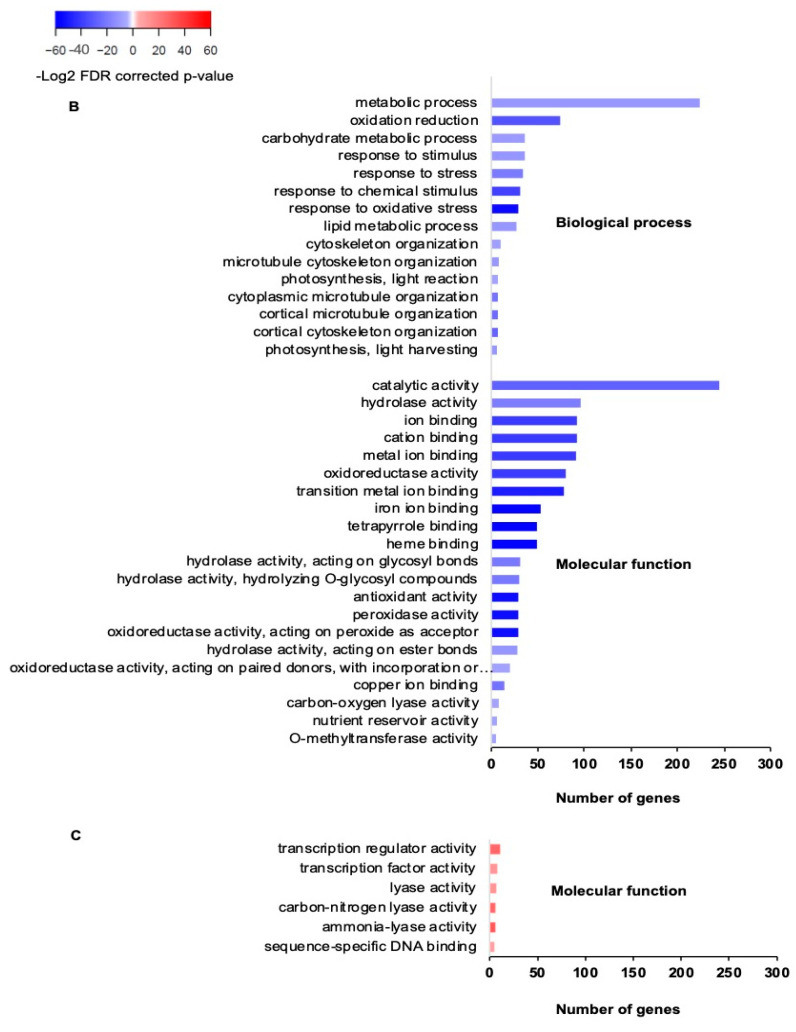
Enriched gene ontology (GO) terms in the two wheat varieties in response to wheat curl mites. GO terms were significantly upregulated in the resistant wheat (**A**), downregulated in the resistant wheat (**B**), and upregulated in the susceptible wheat (**C**). No significantly downregulated enriched GO terms were identified in the susceptible variety. GO terms (FDR corrected *p*-value < 0.05) with significant numbers of upregulated or downregulated genes were identified by contrasting gene expression in response to aphid herbivory and control (aphid-free plants). DEGs were then grouped into biological process, molecular function, and cellular component categories. The colors of the bars refer to the –log2-corrected *p*-values of the corresponding GO terms. The red bars indicate the enriched GO terms in the upregulated genes, and the blue bars indicate the enriched GO terms in downregulated genes. The darker the color, the higher the statistical significance.

A significantly greater number of GO terms was recovered from the resistant wheat exposed to the WCM herbivory compared to that from the susceptible wheat (Figure 4). In the resistant variety, genes associated with several GO terms including metabolic process, biosynthetic process, macromolecule process, and response to abiotic stimulus were significantly upregulated. “Oxidoreductase activity” was also one of the enriched GO terms in the molecular function category in the resistant genotype (Figure 4A). Genes with oxidoreductase activity include cytochrome P450 gene family, peroxidase, lipoxygenase (LOXs), and fatty acid reductase. Lipoxygenase plays an important role in defense responses to biotic and abiotic stresses and also contribute to an antiherbivorous oxidative change on herbivores. This induces oxidative damage to herbivores, both directly and indirectly, and transmits signals that activate plant defenses [44,45]. Increased LOX activity levels have been reported in various interactions between plants and herbivores such as in pigeonpea in response to cotton bollworm, *Helicoverpa armigera* Hübner (Lepidoptera: Noctuidae) [46], and in corn in response to beet armyworm, *Spodoptera exigua* Hübner (Lepidoptera: Noctuidae) herbivory [47]. LOXs are also involved in the activation of downstream pathways including linoleic and linolenic acid and regulating the induction of jasmonic acid (JA) [48,49,50].

Cytochrome P450 superfamily genes were another abundant family of genes with oxidoreductase activity that are induced in response to WCM herbivory. This gene family plays a crucial role in plant defense via multiple biosynthetic and detoxification pathways [51]. Notably, we found 19 genes in this family to be induced in the resistant wheat exposed to WCMs (Supplementary Table S3). Within genes in oxidoreductase activity GO terms, the upregulation of two peroxidase genes, i.e., *Traes_7AL_9040EE6AE* and *Traes_7AL_CE37E2AF1*, were also found in in response to WCMs in the resistant wheat. Peroxidases are an important component of the immediate response of plants to insect damage and involve in deoxidizing radical oxygen species (ROS) enzymes, regulating the redox and Ca^2+^ homeostasis as well as the expression of defense genes [52,53]. Higher expression of peroxidases has been reported previously such as in grape in response to nonadapted strains of two spotted spider mites, *Tetranyhus urticae* Koch (Acari: Tetranchidae) [54], and in pigeonpea in response to cotton bollworm [46]. We also found the upregulation of fatty acid reductase (FAR)-coding genes in response to WCMs in the resistant wheat. It has been reported that *FAR1* improves the resistance to oxidative stress and suppresses plant cell death also by positively regulating the biosynthesis of myo-inositol [55].

A number of DEGs that were downregulated in the resistant wheat exposed to WCMs were categorized as the photosynthesis GO term. The reduction in photosynthesis corroborates previous findings of the effect of herbivory on the reduction of photosynthesis in other species of host plants, such as in cotton in response to two spotted spider mites [56], and in water hyacinth in response to *Orthogalumna terebrantis* Wallwork (Acari: Galumnidae) [57].

On the other hand, very few GO terms were enriched in the susceptible wheat, as expected from a small number of DEGs. Only six GO terms were enriched in upregulated genes within the molecular function category in the susceptible genotype, suggesting a much lower magnitude of transcriptional modification in the susceptible wheat in response to WCM herbivory.

### 2.4. Kyoto Encyclopedia of Genes and Genomes (KEGG) Pathway Analysis

**Figure 5:**
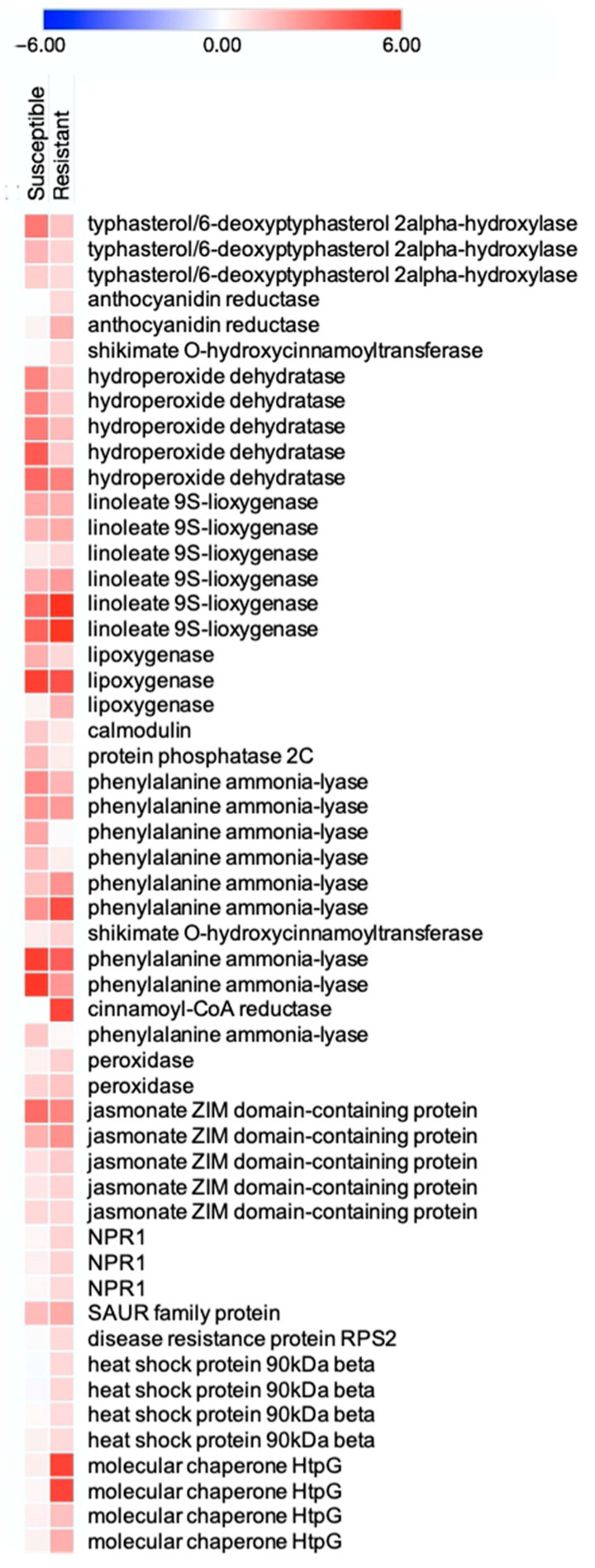
Heat map of DEGs in the resistant and susceptible wheat exposed to wheat curl mites. The heat map shows DEGs across the resistant and susceptible wheat that were mapped to several defense-related pathways. The color key represents log2-transformed fold changes, and the red bars indicate increases in expression in response to wheat curl mite herbivory.

We used BlastKOALA to explore the induction of pathways involved in defense against WCM in the resistant and susceptible wheat. The WCM herbivory induced several pathways including brassinosteroid biosynthesis, flavonoid biosynthesis, linoleic acid metabolism, MAPK signaling pathway, phenylpropanoid biosynthesis, plant hormone signal transduction, and plant pathogen interaction in both wheat varieties, but the number of the upregulated genes in these pathways were higher in the resistant wheat than in the susceptible genotype (Figure 5 and Supplementary Table S3). These pathways are broadly involved in abiotic and biotic stress response in plants and have been shown to be induced by arthropod herbivory in other systems as well. For example, Palmer et al. reported the upregulation of defense-related pathways including phenylpropanoid biosynthesis, flavonoid biosynthesis, and plant-hormone signal transduction in switchgrass in response to fall armyworm, *Spodoptera frugiperda* Smith (Lepidoptera: Noctuidae) [58]. Further, Liang et al. reported the activation of genes involved in signal transduction, flavonoid metabolism, and plant pathogen interactions potentially to have been associated with the resistance of cucumber to *Aphis gossypii* Glover (Hemiptera: Aphididae). [59]. The strong induction of these responses in the resistant genotype suggests a robust deployment of general defense responses in presence of WCMs in this study.

Thirteen genes from the phenylpropanoid pathway, a metabolic pathway responsible for the synthesis of plant secondary metabolites involved in development and stress responses, were induced in response to WCMs in both wheat varieties. However, a significantly higher number of genes involved in this pathway were induced in the resistance wheat. These genes included transcripts coding for phenylalanine ammonia-lyase (PAL), peroxidases, shikimate O-hydroxycinnamoyl transferases, and cinnamoyl-CoA reductase that are known to play a role in biotic stresses defense responses [60,61]. Specifically, eight genes encoding PAL showed more than 2-fold increase in response to WCM herbivory in the resistant wheat (Supplementary Table S3). PAL catalyzes the first step of the phenylpropanoid pathway and converts L-phenylalanine to trans-cinnamate and ammonia, which is a key reaction in the control of lignin, flavonoid, and salicylic acid biosynthesis. Several studies have previously illustrated the effects of *PAL* knockdown on plant growth and response to herbivory. For example, Van Eck et al. reported that *PAL* silencing increased wheat susceptibility to Russian wheat aphids, *Diuraphis noxia* Mordvilko (Hemiptera: Aphididae) [62]. It has been suggested that reduced PAL can have a positive effect on the supply of carbohydrates but an adverse effect on disease resistance. Lv et al. reported that the expression of PAL was significantly increased in cotton and corn seedlings damaged by mechanical wounding as well as cotton aphid and corn borer *Ostrinia furnacalis* Guenée (Lepidoptera: Crambidae) herbivory [63]. The induction of these genes in our experiment suggests that resistant wheat can mount broad antioxidative responses, which are lacking in the susceptible variety.

Three nonexpressors of pathogenesis-related genes 1 (*NPR1*) were also induced in response to WCMs in the resistant wheat. The transcription co-activator NPR1 is the master regulator of SA signaling and interacts with transcription factors to induce the expression of antimicrobial pathogenesis-related (PR) genes. Five DEGs in the JA signaling pathway that regulates direct and indirect plant responses against herbivores [64,65,66] were also induced in our experiment. These DEGs belong to the jasmonate ZIM domain (JAZ) family and showed more than 2-fold change in response to WCM herbivory in the resistant variety but did not show differential expression in the susceptible variety (Figure 5). JAZ acts as a JA signal output repressor. High levels of JAZ proteins regulate JA reactions by suppressing their transcription factors [65,67]. JAZ proteins have been reported to help balance the growth–defense continuum by matching the level of biotic stress to available resources [68].

Further, four heat shock protein 90KDA (Hsp90) were upregulated in the resistant wheat in response to WCM herbivory. Heat shock proteins play a role in stress signal transduction and help stabilize protein structures and DNA repair by modulating DNA repair complex architecture and regulating cellular redox state [69,70]. The majority of Hsp90 substrates are kinases and transcription factors, which function primarily in signal transduction to activate or suppress the expression of the defense gene generation and in the control of the interaction between different signaling pathways [71] We noted significant upregulation of four Hsp90 genes in the resistant wheat, which was lacking in the susceptible genotype. Changes in expression patterns of these genes likely contributed to improved resistance to WCMs. It has been reported that knocking down Hsp90 weakens the *Mi-1*-mediated resistance to root-knot nematode and potato aphid in tomato [72].

### 2.5. RT-qPCR of the Selected DEGs

Strong correlations were found between RNA-seq and RT-qPCR results (Supplementary Figure S1), indicating that the measured changes in gene expression detected by RNA-seq reflected the actual transcriptome differences between the samples.

## 3. Materials and Methods

### 3.1. Plant Materials and WCM Colonies

All experiments were conducted at the Texas A&M AgriLife Research greenhouse complex located at the Plant Stress Laboratory in Bushland, TX. Two wheat varieties, WCM-susceptible (Karl 92) and WCM-resistant (TAM112) [38], both at 2-week postemergence and grown under a long-day photoperiod (16L:8D), were used in this study. The two varieties were grown under controlled greenhouse conditions with a temperature range of 21 ± 3 °C (night) to 30 ± 3 °C (day). Plants were grown in pots containing Redi-earth plug and seedling soil mix (Sun Grow Horticulture Inc., Agawam, MA, USA) in insect-proof rearing tents with a dimension of 60 cm × 60 cm × 60 cm (MegaView Science Education Services Co., Taipei, Taiwan). The WCMs used in the study were from a colony maintained by the Plant Pathology Program held by Texas A&M AgriLife Research Center in Bushland, Texas.

### 3.2. Sample Collection, RNA Isolation, and Sequencing

The experiment was a factorial design with two levels of the wheat variety factor (susceptible and resistant) and two levels of WCM herbivory (WCM present and WCM absent). Each treatment combination was replicated four times (*n* = 16). Two weeks after germination at one shoot stage, half of the susceptible and half of the resistant plants were randomly assigned to the herbivory treatment. Plants assigned to the WCM herbivory treatment were infested using a wheat leaf clipping measuring approximately 1 cm and containing approximately 20 WCMs, which was placed in the leaf whorl of the test plants. Wheat clippings free of WCMs were placed inside whorls of wheat assigned to the WCM-absent treatment. Twenty-four h following WCM infestation, the entire above-ground tissue of each plant regardless of treatment was collected for RNA-seq. The samples were immediately frozen in liquid nitrogen (LN_2_) and stored at −80 °C until further sample processing. The frozen leaf samples were ground under LN_2_ into a fine powder using a mortar and pestle. RNA was extracted from 100 mg of frozen ground tissue using the miRNeasy Mini Kit (Qiagen, Valencia, CA, USA) and subsequently treated with the DNase Max Kit (Qiagen, Valencia, CA, USA). RNA quality and quantity were assessed using the NanoVue Plus spectrophotometer (GE, Healthcare, Piscataway, NJ, USA). Three equimolar RNA samples extracted from three technical replicates for each biological replicate were pooled after extraction for RNA-seq template preparation. The quality of each pooled RNA sample was assessed with the Agilent 2100 Bioanalyzer (Agilent Technologies, Santa Clara, CA, USA) prior to RNA-seq template preparation by Texas AgriLife Research Genomic and Bioinformatics Services. Paired-end (PE) sequencing was performed on an Illumina HiSeq4000 using a 150 bp paired-end strategy on four biological replicates. RNA-seq template preparation and sequencing was completed at Texas AgriLife Research Genomics and Bioinformatics Center.

### 3.3. Gene Expression Analysis

Sequence reads were imported into the CLC Genomics Workbench version 20 (Qiagen, Valencia, CA, USA) and mapped to two versions of *Triticum aestivum* reference genomes, including *Triticum aestivum* v2.2 (www.phytozome.jgi.doe.gov; accessed on 6 January 2020 [39]) and IWGS RefSeq v.1.0 (https://plants.ensembl.org; accessed on 6 January 2020 [40]). Based on the total read counts for each annotated gene, differential gene expression analyses were conducted using the Empirical Analysis of the DGE tool, which implements the “Exact Test” for two group comparisons [41]. Expression levels of genes in response to mite herbivory were compared to the mite-free plants (the control) samples of the same variety to identify DEGs. The DEGs were defined as having a fold change of ≥1.9 or ≤−1.9 with a false discovery rate (FDR)-corrected *p*-value of <0.05. For functional annotation, GO was performed using AgriGO gene ontology analysis tools [42]. To underline the pathways in which the DEGs contribute ti BlastKOALA was used to search the KEGG database [43]. To further investigate the transcriptome changes in response to mite herbivory, MapMan software [44] was used to analyze differential expression in metabolic pathways. Heat maps were generated using Morpheus (https://software.broadinstitute.org/morpheus; accessed on 7 July 2020).

### 3.4. RT-qPCR Analysis of the Selected DEGs

For the validation of RNA-seq data using RT-qPCR, five DEGs were selected. The same RNA samples from four biological replicates that were used for the sequencing were used for RT-qPCR. The total RNA was reverse-transcribed using the Omniscript RT Kit (QIAGEN, Valencia, CA, USA). Primer pairs were designed using NCBI Primer-BLAST (http://www.ncbi.nlm.nih.gov/tools/primer-blast/; accessed on 9 January 2020), which are listed in Supplementary Table S4. The RT-qPCR reaction was performed in a total volume of 20 μL, containing 1 μL of diluted cDNA, 0.5 μL of reverse and forward primers, 8 μL of ddH2O, and 10 μL of SYBR Green Master Mix (Applied Biosystems, Foster City, CA, USA). The samples were run on an Applied Biosystems ViiA 7 Real-Time PCR system (Applied Biosystems, Foster City, CA, USA) according to the standard protocol as follows: 2 min at 50 °C, 10 min at 95 °C, followed by 40 cycles for 15 s each at 95 °C, and 1 min at 60 °C. A melt curve was generated by the end of each PCR reaction to verify the formation of a single peak and to exclude the possibility of primer dimer and nonspecific product formation. All reactions were performed in duplicate, including nontemplate control reactions. The relative quantitation of gene expression of RT-qPCR was measured using the 2^−ΔΔCt^ method [73], with Ta2776 (RNase L inhibitor-like protein) gene [74] as the endogenous reference gene that showed the highest stability among three tested reference genes. The correlation between the RNA-seq data and the RT-qPCR results was determined by the Pearson’s correlation coefficient.

## 4. Conclusions

Despite decades of research and selection for resistance against WCMs and associated viruses, we know little of the molecular mechanisms that underlie WCM–wheat interactions. Strengthening innate plant defenses is essential to sustainable suppression of this pest and mitigating the impact of viruses associated with it to wheat production. This is the first study to explore how resistant wheat modulates its transcriptional responses when exposed to WCM herbivory and provides important insights into mechanisms that contribute to enhancing host plant resistance.

We noted a significant induction of a suite of genes and pathways involved in general plant responses to biotic and abiotic stress in the resistant wheat that was absent in the wheat susceptible to the mites. These transcriptome modifications in resistant plants included the expression of genes involved in JA defense pathways, WKRY transcription factors, antioxidation processes, and pathogen-related responses, among others. Susceptible plants, on the other hand, appeared to undergo few, if any, relevant transcription modifications when exposed to WCM herbivory. The ability of resistant plants to alter their transcriptomes to respond to an herbivore is a common theme across many plant systems, providing evidence that the plasticity of molecular responses that follow herbivory is key to the effective induction of defenses. However, the exact mechanisms that trigger these responses are not clear, and additional research is needed to uncover the defense on- and off-switches that allow resistant host plants to recognize and respond to herbivores. Further, WCM associations with a suite of different plant viruses is likely to affect the outcome of these interactions, and research focused on understanding how virus presence alters wheat responses to mites will be key to the development of effective host plant resistance.

## Data Availability

All raw sequencing reads have been submitted to the NCBI Sequence Read Archive and are available under BioProject ID: PRJNA693542.

## Supplementary Materials

Supplementary materials can be found at https://www.mdpi.com/1422-0067/22/5/2703/s1. Figure S1: Real-time quantitative RT-PCR (RT-qPCR) validation of gene expression changes, Table S1: Summary of RNA-seq reads from the susceptible and resistant wheat varieties mapped to the wheat genome (*Triticum aestivum* v2.2), Table S2: Summary of RNA-seq reads from susceptible and resistant wheat varieties mapped to the wheat genome (IWGSC RefSeq v.1.0). Table S3: List of differentially expressed genes (DEGs) that were mapped to several defense-related pathways in the resistant and susceptible wheat exposed to wheat curl mites. Table S4: Primers sequences used for the RT-qPCR amplification of the five DEGs selected for validation.
