# Supplementary material for: Transcriptional Responses of Resistant and Susceptible Wheat Exposed to Wheat Curl Mite
Source: Int J Mol Sci. 2021 Mar 8;22(5):2703. doi: 10.3390/ijms22052703 (PMC7962190; doi:10.3390/ijms22052703)
Supplement: Supplementary file 1 [file ijms-22-02703-s001.zip › Supplementary-files/Supplementary Table S4.docx]

**Supplementary Table S4 : Primers sequences used for RT-qPCR amplification of the five differentially expressed genes selected for validation.**

| Gene ID | Primers sequences |
| --- | --- |
| \| ***Ta2776*** \| *AGTTGGTCGGGTCTCTTCTAAATG* \| \| --- \| --- \| | F:5’-CGATTCAGAGCAGCGTATTGTTG-3’ |
|  | R:5’-AGTTGGTCGGGTCTCTTCTAAATG-3’ |
| *Traes_3B_02B5D826F* | F:5'-GGTTGATGCTGCCATCGCTA-3' |
|  | R:5'- GCAGTCTATCAGCCAGACCGA-3' |
| *Traes_4BL_523D155E21* | F:5'- TTCGGCGTGCTCCCTTCTG-3' |
|  | R:5'- CTGGAATATGAGCCACTTTGCG-3' |
| *Traea_4BL_EB9CF2010* | F:5'- TCAGTCAGGCTGTCTAGTGC-3' |
|  | R:5'- ACAAGGTGGACTCGAACACAG-3' |
| *Traes_6AS_F061F607E* | F:5'- AGGATATGGGCATCACCGTC-3' |
|  | R:5'- AACGTCAGAGACAAAGGACTG-3' |
| *Traes_7DS_9B1077F7D* | F:5'- GTCGTCGTCTGTCCACCTAC-3' |
|  | R:5'- GGAGCCTCCAGTCATTCCAC-3' |
