# Supplementary material for: Transcriptional Responses of Resistant and Susceptible Wheat Exposed to Wheat Curl Mite
Source: Int J Mol Sci. 2021 Mar 8;22(5):2703. doi: 10.3390/ijms22052703 (PMC7962190; doi:10.3390/ijms22052703)
Supplement: Supplementary file 1 [file ijms-22-02703-s001.zip › Supplementary-files/Supplementary Table S2.docx]

## **Supplementary Table S2: Summary of RNA-seq reads from susceptible and resistant wheat varieties mapped to the wheat genome (**IWGSC RefSeq v.1.0). Unique RNA-seq reads mapping to exons, introns, and intergenic regions are shown as the percentage of total reads distributed to these annotated regions of the wheat genome.

| **Genotype** | **Treatment** | **Rep number** | **Total reads (M)** | **Mapped reads (M)** | **Exonic reads (%)** | **Intronic reads (%)** | **Intergenic reads (%)** |
| --- | --- | --- | --- | --- | --- | --- | --- |
| **Susceptible** | +Mite | 1 | 98.24 | 75.36 | 81.55 | 5.96 | 12.48 |
| **Susceptible** | +Mite | 2 | 77.98 | 58.32 | 80.19 | 6.08 | 13.73 |
| **Susceptible** | +Mite | 3 | 72.88 | 54.39 | 81.30 | 5.97 | 12.73 |
| **Susceptible** | +Mite | 4 | 78.53 | 59.11 | 77.13 | 6.28 | 16.59 |
| **Mean ± sd** |  |  | 81.90 ± 11.18 | 61.79 ± 9.27 | 80.04 ± 2.02 | 6.07 ± 0.14 | 13.88 ± 1.88 |
| **Susceptible** | Control | 1 | 74.17 | 58.57 | 81.41 | 6.04 | 12.55 |
| **Susceptible** | Control | 2 | 67.60 | 54.38 | 81.18 | 6.27 | 12.55 |
| **Susceptible** | Control | 3 | 72.34 | 57.31 | 80.97 | 6.19 | 12.84 |
| **Susceptible** | Control | 4 | 79.24 | 58.66 | 80.91 | 6.22 | 12.87 |
| **Mean ± sd** |  |  | 73.33 ± 4.81 | 57.23 ± 1.99 | 81.11 ± 0.22 | 6.18 ± 0.09 | 12.70 ± 0.17 |
| **Resistant** | +Mite | 1 | 84.28 | 59.90 | 71.83 | 6.87 | 21.30 |
| **Resistant** | +Mite | 2 | 77.47 | 57.01 | 71.59 | 6.82 | 21.59 |
| **Resistant** | +Mite | 3 | 76.77 | 55.80 | 72.37 | 6.52 | 21.10 |
| **Resistant** | +Mite | 4 | 82.23 | 55.12 | 72.13 | 6.59 | 21.28 |
| **Mean ± sd** |  |  | 80.18 ± 3.65 | 56.95 ± 2.11 | 71.98 ± 0.34 | 6.70 ± 0.17 | 21.31 ± 0.20 |
| **Resistant** | Control | 1 | 76.17 | 57.18 | 74.56 | 6.52 | 18.92 |
| **Resistant** | Control | 2 | 77.87 | 60.86 | 73.77 | 6.61 | 19.62 |
| **Resistant** | Control | 3 | 80.89 | 56.42 | 72.11 | 6.64 | 21.24 |
| **Resistant** | Control | 4 | 75.63 | 56.98 | 75.54 | 6.51 | 17.95 |
| **Mean ± sd** |  |  | 77.64 ± 2.36 | 57.86 ± 2.02 | 73.99 ± 1.45 | 6.57 ± 0.06 | 19.43 ± 1.38 |
