# Supplementary material for: Transcriptional Responses of Resistant and Susceptible Wheat Exposed to Wheat Curl Mite
Source: Int J Mol Sci. 2021 Mar 8;22(5):2703. doi: 10.3390/ijms22052703 (PMC7962190; doi:10.3390/ijms22052703)
Supplement: Supplementary file 1 [file ijms-22-02703-s001.zip › Supplementary-files/Supplementary Table S1.docx]

## **Supplementary Table S1: Summary of RNA-seq reads from susceptible and resistant wheat varieties mapped to the wheat genome (***Triticum aestivum* v2.2**).** Unique RNA-seq reads mapping to exons, introns, and intergenic regions are shown as the percentage of total reads distributed to these annotated regions of the wheat genome.

| **Genotype** | **Treatment** | **Rep number** | **Total reads (M)** | **Mapped reads (M)** | **Exonic reads (%)** | **Intronic reads (%)** | **Intergenic reads (%)** |
| --- | --- | --- | --- | --- | --- | --- | --- |
| **Susceptible** | +Mite | 1 | 98.24 | 63.14 | 91.56 | 3.15 | 5.29 |
| **Susceptible** | +Mite | 2 | 77.98 | 48.55 | 90.84 | 3.34 | 5.82 |
| **Susceptible** | +Mite | 3 | 72.88 | 45.20 | 91.12 | 3.25 | 5.63 |
| **Susceptible** | +Mite | 4 | 78.53 | 48.44 | 88.48 | 3.26 | 8.27 |
| **Mean ± sd** |  |  | 81.90 ± 11.18 | 51.33 ± 8.02 | 90.50 ± 1.37 | 3.25 ± 0.07 | 6.25 ± 1.36 |
| **Susceptible** | Control | 1 | 74.17 | 48.51 | 91.55 | 3.38 | 5.07 |
| **Susceptible** | Control | 2 | 67.60 | 44.92 | 91.61 | 3.44 | 4.95 |
| **Susceptible** | Control | 3 | 72.34 | 47.47 | 91.31 | 3.38 | 5.30 |
| **Susceptible** | Control | 4 | 79.24 | 49.59 | 91.22 | 3.51 | 5.27 |
| **Mean ± sd** |  |  | 73.33 ± 4.81 | 47.62 ± 1.99 | 91.42 ± 0.18 | 3.42 ± 0.06 | 5.14 ± 0.16 |
| **Resistant** | +Mite | 1 | 84.28 | 46.12 | 87.69 | 3.60 | 8.72 |
| **Resistant** | +Mite | 2 | 77.47 | 43.15 | 86.24 | 3.46 | 10.30 |
| **Resistant** | +Mite | 3 | 76.77 | 43.67 | 85.44 | 3.06 | 11.50 |
| **Resistant** | +Mite | 4 | 82.23 | 46.31 | 79.25 | 3.01 | 17.74 |
| **Mean ± sd** |  |  | 80.18 ± 3.65 | 44.81 ± 1.63 | 84.65 ± 3.72 | 3.28 ± 0.29 | 12.06 ± 3.95 |
| **Resistant** | Control | 1 | 76.17 | 45.90 | 86.09 | 3.13 | 10.78 |
| **Resistant** | Control | 2 | 77.87 | 46.09 | 88.54 | 3.19 | 8.27 |
| **Resistant** | Control | 3 | 80.89 | 44.74 | 83.47 | 3.16 | 13.37 |
| **Resistant** | Control | 4 | 75.63 | 45.82 | 86.14 | 3.17 | 10.69 |
| **Mean ± sd** |  |  | 77.64 ± 2.36 | 45.63 ± 0.60 | 86.06 ± 2.07 | 3.16 ± 0.02 | 10.77 ± 2.08 |
