# Supplementary material for: Transcriptional Responses of Resistant and Susceptible Wheat Exposed to Wheat Curl Mite
Source: Int J Mol Sci. 2021 Mar 8;22(5):2703. doi: 10.3390/ijms22052703 (PMC7962190; doi:10.3390/ijms22052703)
Supplement: Supplementary file 1 [file ijms-22-02703-s001.zip › Supplementary-files/Supplementary Figure S1.pdf]

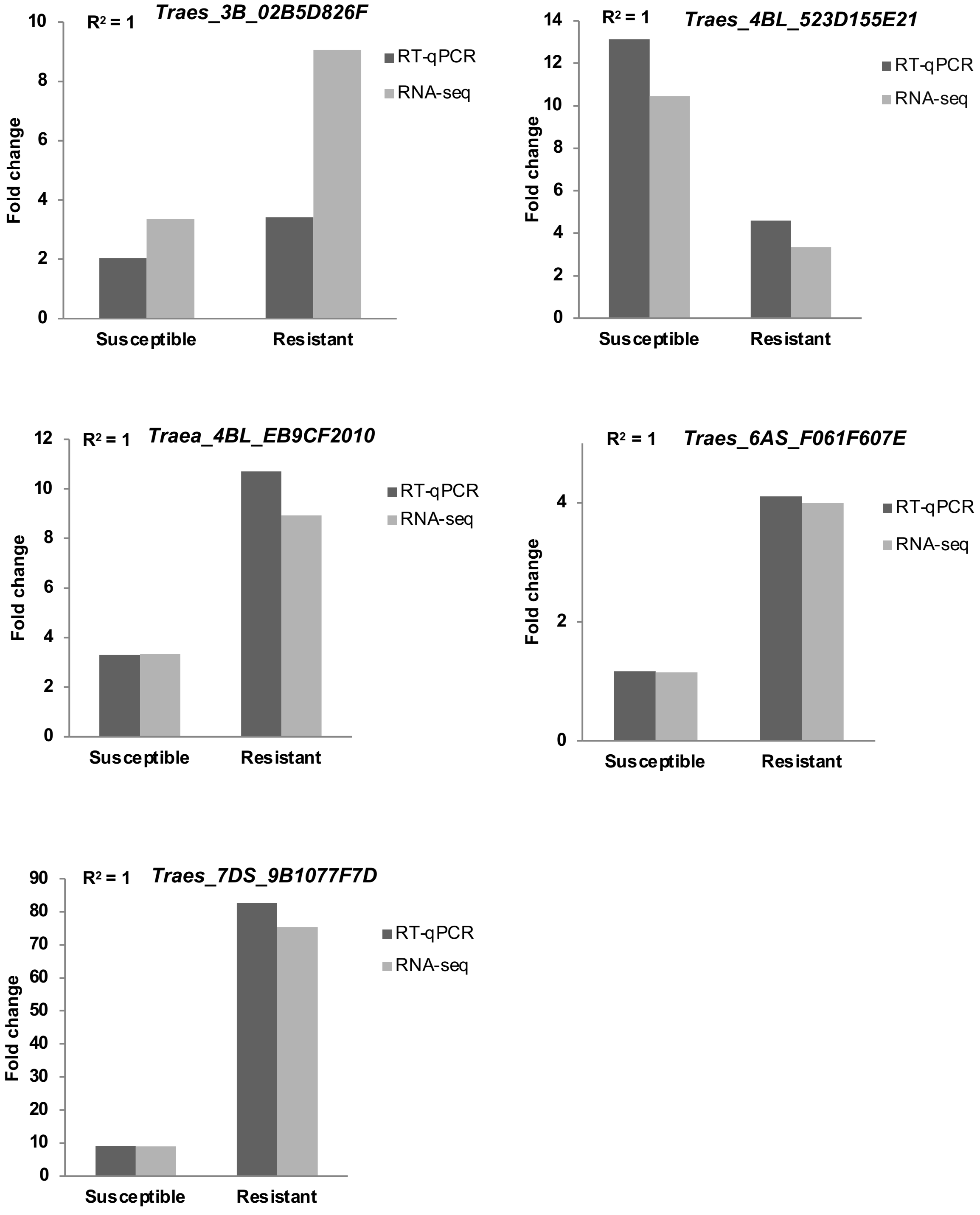

Supplementary Figure S1: Real-time quantitative RT-PCR (RT-qPCR) validation of gene expression changes detected by RNA-seq. Data are shown as a fold change of the mean expression levels from three biological replicates. The correlation between the RNA-seq data and the RT-qPCR results is shown by Pearson's correlation coefficient (R2).
